# Stability and ocular biodistribution of topically administered PLGA nanoparticles

**DOI:** 10.1038/s41598-021-90792-5

**Published:** 2021-06-10

**Authors:** Sean Swetledge, Renee Carter, Rhett Stout, Carlos E. Astete, Jangwook P. Jung, Cristina M. Sabliov

**Affiliations:** 1 Department of Biological and Agricultural Engineering, Louisiana State University, Baton Rouge, LA 70803 USA; 2 Veterinary Clinical Sciences, Louisiana State University and LSU Veterinary Medicine, Skip Bertman Drive, Baton Rouge, LA 70803 USA; 3 Pathobiological Sciences, Louisiana State University and LSU Veterinary Medicine, Skip Bertman Drive, Baton Rouge, LA 70803 USA; 4 Department of Biological and Agricultural Engineering, Louisiana State University and LSU Agricultural Center, Baton Rouge, LA 70803 USA

**Keywords:** Medical research, Engineering, Materials science

## Abstract

Polymeric nanoparticles have been investigated as potential delivery systems for therapeutic compounds to address many ailments including eye disease. The stability and spatiotemporal distribution of polymeric nanoparticles in the eye are important regarding the practical applicability and efficacy of the delivery system in treating eye disease. We selected poly(lactic-co-glycolic acid) (PLGA) nanoparticles loaded with lutein, a carotenoid antioxidant associated with eye health, as our model ophthalmic nanodelivery system and evaluated its stability when suspended in various conditions involving temperature and light exposure. We also assessed the ocular biodistribution of the fluorescently labeled nanoparticle vehicle when administered topically. Lutein-loaded nanoparticles were stable in suspension when stored at 4 °C with only 26% lutein release and no significant lutein decay or changes in nanoparticle morphology. When stored at 25 °C and 37 °C, these NPs showed signs of bulk degradation, had significant lutein decay compared to 4 °C, and released over 40% lutein after 5 weeks in suspension. Lutein-loaded nanoparticles were also more resistant to photodegradation compared to free lutein when exposed to ultraviolet (UV) light, decaying approximately 5 times slower. When applied topically in vivo, Cy5-labled nanoparticles showed high uptake in exterior eye tissues including the cornea, episcleral tissue, and sclera. The choroid was the only inner eye tissue that was significantly higher than the control group. Decreased fluorescence in all exterior eye tissues and the choroid at 1 h compared to 30 min indicated rapid elimination of nanoparticles from the eye.

## Background

Various treatment methods have been used to deliver pharmaceuticals to the posterior of the eye, including topical, systemic and intravitreal routes. However, the protective barriers within the eye limit the efficiency and effectiveness of these treatments^1^. Advancements in nanoparticle therapeutics for ocular drug delivery have provided an alternative treatment option, which enhances spatiotemporal control of drug delivery and can exhibit stimuli-responsive behavior such as thermos-responsive gelling or pH-triggered drug release with advanced formulations^2,3^. Polymeric nanoparticle drug delivery systems have been tested for the treatment of various eye diseases including cataracts^4^, glaucoma^5^, corneal graft rejection^6^, and autoimmune uveitis^7^. Nanodelivery systems are a pivotal tool to enhance current disease therapies by improving topical passage of large hydrophobic drugs, increasing drug contact time with the target tissue, and improving targeted delivery of drugs to specific ocular tissues^8^.

The two most common routes for nanodelivery of drugs to the eye are intravitreal and topical, with intravitreal injections being the preferred route of administration in treating posterior eye diseases^9^. Although intravitreal injections are preferred when treating posterior eye diseases due to quick and direct tissue targeting, repeated injections lead to an increased incidence of complications, such as endophthalmitis, retinal detachment, and intraocular hemorrhag^10^. Therefore, topical application of drugs to target posterior tissues has received increasing attention due to its improved patient compliance, reduced systemic toxicity, non-invasiveness, and decreased risk of ocular complications^11^. However, eye drops have very poor ocular bioavailability due to rapid tear turnover rate and impermeability of drugs through the corneal epithelium, causing only about 5% of lipophilic drugs and less than 0.5% of hydrophilic drugs to reach intraocular tissues^12,13^. In contrast, when drugs are entrapped and delivered by nanoparticles, their intraocular bioavailability is dramatically improved by increasing the amount of drug that can be added and contact time with the cornea^14^. Many reports showed that nanoparticles can overcome many ocular barriers by engineering surface properties and hydrophobicity^15^.

Lutein is the primary component of macular pigment in the retina with antioxidant properties known to protect the eye from photo-oxidative stress. The three-peak absorbance of lutein ranges from 425–480 nm^16^, which can contribute to the protection of the retina by shielding DNA and cytochrome^17,18^. The therapeutic efficacy of nanodelivered lutein has been demonstrated by Bodoki et al.^4^, finding that topically applied, lutein-loaded polymeric nanoparticles significantly attenuated cataract development in a rat model more than free lutein and oral formulations. Unfortunately, lutein is relatively unstable and will degrade at higher temperatures or when directly exposed to light. Thus, loading lutein into nano-formulations simultaneously addresses its poor stability and hydrophobicity, allowing it to resist degradation and disperse in aqueous solutions. Storage temperature, resistance to photo- and thermal-degradation, release kinetics, and biodistribution of nanoparticle-based lutein therapeutics in the eye are important considerations to optimize treatment effectiveness. Here, we assessed temperature-dependent physical stability of PLGA nanoparticles, thermal stability, photostability and release of lutein from PLGA nanoparticles, and time-dependent ocular biodistribution of topically applied Cy5-labeled PLGA nanoparticles.

## Methods

### Nanoparticle synthesis

Cy5 fluorescently labeled PLGA nanoparticles were synthesized using the emulsion evaporation technique. A 2% (w/v) solution of Polyvinyl Alcohol (PVA) (31–50 kDa) was prepared in water by dissolving PVA at 60 °C. The organic phase was prepared by dissolving 400 mg 50:50 PLGA (24–38 kDa) and 15 mg cyanine-5 (Cy5) dye (Lumiprobe, Hallandale Beach, FL, USA) in 8 mL ethyl acetate. For lutein-loaded nanoparticles, the organic phase consisted of 40 mg lutein (Kemin Foods L.C., Des Moines, IA, USA) dissolved in 8 mL ethyl acetate. The aqueous phase was a 100 mL solution of Tween-80 (4.5 mg/mL) in deionized water and was saturated with 10 mL ethyl acetate. The organic phase was added dropwise to the aqueous phase and allowed to stir for 10 min. The emulsion was passed through a microfluidizer (M-11 OP, Microfluidics, MA, USA) 4 times at 30 kpsi. Ethyl acetate was removed by rotary evaporation for 90 min. Trehalose monohydrate was added (1100 mg) as a cryoprotectant, followed by 10 mL of the PVA solution. The nanoparticle suspension was frozen at -80 °C, freeze-dried (Labconco, USA) for 48 h, and the nanoparticle powder was stored at -20 °C until use.

### Nanoparticle characterization

Freeze-dried nanoparticles were resuspended in water and analyzed by transmission electron microscopy (TEM) (JEOL-4000, Tokyo, Japan) and dynamic light scattering (DLS) using a Malvern Zetasizer (Malvern, United Kingdom) for size, polydispersity, and morphology. To measure lutein entrapment efficiency, freeze-dried nanoparticles were resuspended in water (5 mg/mL). Total lutein was extracted from 100 µL aliquots of the suspension with 900 µL acetonitrile (30 min incubation). Non-entrapped lutein was separated from nanoparticles in the suspension via centrifugation (30 min, 4 °C, 64,000 RCF), and lutein was extracted from the supernatant as described above. Samples were measured in triplicate for absorbance at 445 nm (Cytation 3, BioTek, Winooski, VT, USA), and the non-entrapped lutein was divided by the total lutein and subtracted by 100% to obtain the entrapment efficiency.

Lutein-loading was also confirmed via X-Ray diffraction (XRD). The Panalytical Empyrean (Malvern Panalytical Inc., Westborough, MA) multipurpose diffractometer equipped with PreFIX (pre-aligned, fast interchangeable X-ray) modules was used to obtain the spectrums of the sample powders. The method used was the theta/2theta scan operating with anode material of Cu, K-alpha1 and K-alpha2 radiation with a ratio of K-alpha2/K-alpha1 of 0.5. Generator voltage of 45 and tube current 40. The scans performed ranged from 4.98° to 60°.

### Physical stability of nanoparticles

Freeze-dried nanoparticles were resuspended at 5 mg/mL and aliquoted into vials that were stored at 4 °C, 25 °C, and 37 °C. At weekly timepoints, samples were removed and diluted to 0.2 mg/mL for DLS analysis and 1 mg/mL for (TEM).

### Lutein release from nanoparticles

Freeze-dried nanoparticles were resuspended in PBS (pH 7.4) at 5 mg/mL and loaded into dialysis tubing (12–14 KDa, Thermo Fisher Scientific, Waltham, MA, USA) which was immersed in 1L PBS (pH 7.4) and constantly stirring. Release was measured at 4 °C, 25 °C, and 37 °C in triplicate, and the PBS outside the tubing was changed every 48 h. At predetermined timepoints, 100 µL was collected from inside the dialysis tubing, mixed with 900 µL acetonitrile, and stored at 4° C until measured via absorbance at 445 nm (Cytation3).

### Lutein thermal stability in nanoparticles

Lutein thermal stability was measured at different temperatures. Freeze-dried nanoparticles were resuspended in PBS (pH 7.4) at 5 mg/mL. The suspension was aliquoted into vials for each timepoint in triplicate (0.5 mL) which were stored at 4 °C, 25 °C, and 37 °C. At predetermined timepoints, 100 µL was collected from designated vials, mixed with 900 µL acetonitrile, and shaken for 30 min. The samples were then measured for absorbance at 445 nm.

### Nanoparticle enhancement of lutein photostability

Lutein photo-stability was assessed under UV light. Freeze dried nanoparticles were resuspended in water at 5 mg/mL, and an equivalent amount of free lutein (95.7 µg/mL) was dissolved in dimethyl sulfoxide (DMSO). The nanoparticle suspension and free lutein solution were aliquoted into microcentrifuge tubes and placed under a UV floodlight (Intelliray 400, Uvitron International, West Springfield, MA, USA) at 50% intensity and 10.5 in exposure height for 6 h. At predetermined timepoints, 100 µL of samples were taken, mixed with 900 µL acetonitrile, incubated at room temperature for 30 min, and read for absorbance at 445 nm.

### Biodistribution pilot study

A pilot study was conducted to measure the biodistribution of fluorescent nanoparticles in the eyes of mice in vivo*.* The nanoparticle synthesis techniques and treatment preparation are described in supplementary material. Courmain-6 loaded PEG-PLGA nanoparticles were suspended in either saline or a thermosensitive hydrogel and applied topically to the left eyes of mice, while only saline or the hydrogel was applied to the right eye as a control. Rats were divided into 4 groups consisting of the two suspension mediums and two timepoints post-application: 30 min and 60 min. Methods for sample processing, image analysis, and statistical analysis are described in supplementary material. The study was carried out in compliance with the ARRIVE guidelines.

### In vivo biodistribution

Animals were housed and treated according to the Louisiana State University animal care policies and in compliance with the American Veterinary Medical Association (AVMA). The experimental protocols were approved by Louisiana State University Institution Animal Care and Use Committee (IACUC).

Adult (37–46 weeks old) Wistar rats (Envigo, Huntingdon, United Kingdom) were divided randomly into four groups: three experimental groups which received the same dose of fluorescent nanoparticles but were euthanized at different times (15, 30, and 60 min after application), and one control group which received PBS (control). The treatment was prepared by suspending dry nanoparticles in PBS at 20 mg/mL. One drop of either the nanoparticle suspension or PBS, approximately 12 µL, was applied to both eyes of each animal. Controls and treated animals were kept separate during the incubation period to prevent cross contamination of treatments between animals. At different time points tested, animals were euthanized in a CO_2_ gas chamber. The eyes were immediately enucleated after euthanasia, immersed in OCT freezing media, and snap frozen in isopentane cooled by liquid nitrogen. Eyes were cryo-sectioned (Thermo Shandon Cryotome E, Cambridge, UK) at 8 μm and imaged using an Eclipse Ti2 microscope (Nikon Instruments Inc., Melville, NY, USA). Both phase-contrast and fluorescence images were taken to capture tissue morphologies and fluorescence from nanoparticles.

### Image analysis

**Figure 1:**
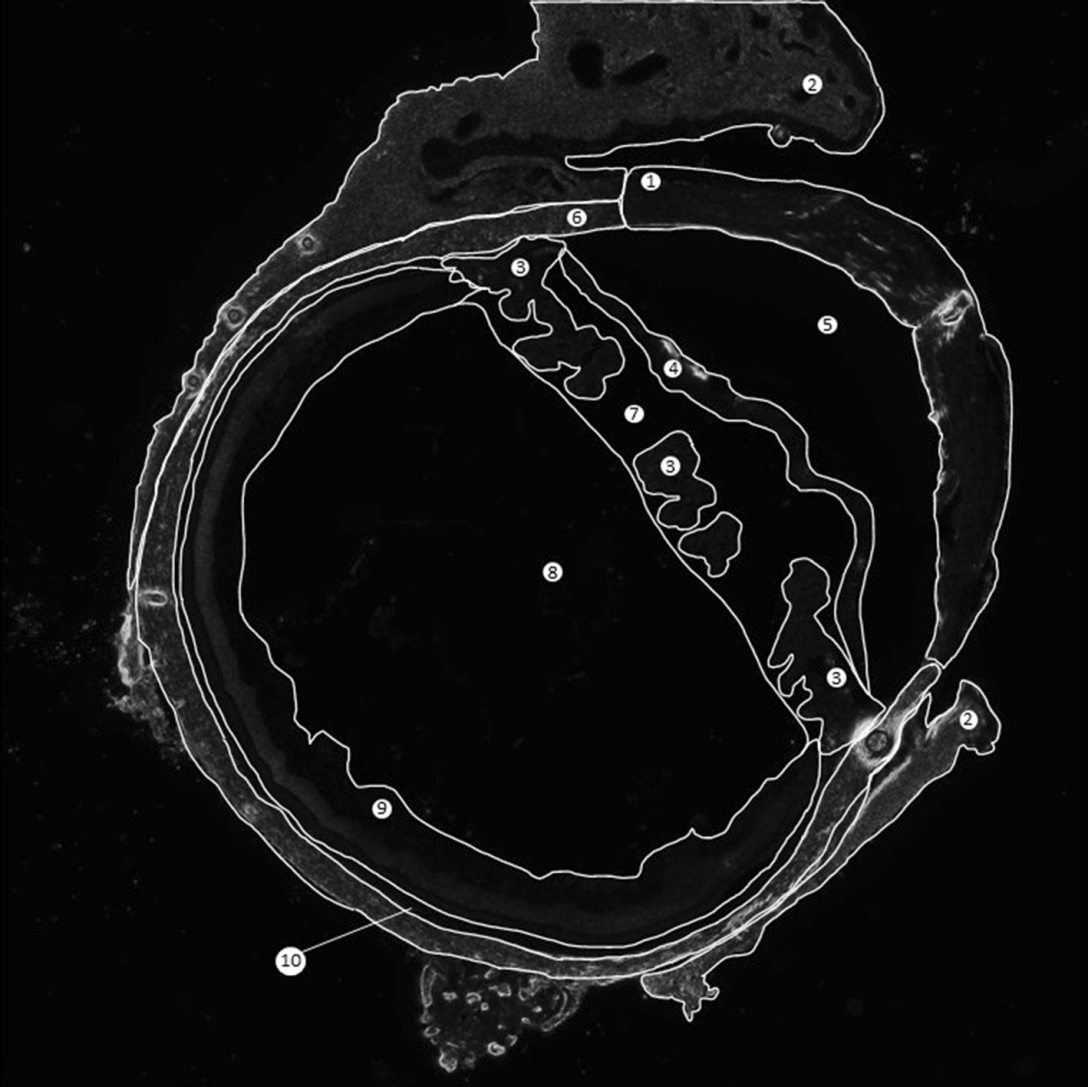
Regions of interest analyzed. Labels are as follows: (1) Cornea, (2) Episcleral Tissue, (3) Ciliary Body, (4) Iris, (5) Anterior Chamber, (6) Sclera, (7) Posterior Chamber, (8) Vitreous, (9) Retina (artificial detachment due to sectioning), (10) Choroid.

Fiji software was used to quantify fluorescence intensity in the eye as a total, and individual structures of the eye: cornea, conjunctiva, iris and ciliary body, lens, sclera, retina, and exterior eye muscle. For individual eye structure analysis, a region of interest (ROI) was drawn around each structure and analyzed. To aid in identifying the tissue boundaries and drawing the ROI, bright field images were overlaid on fluorescence images of the same sample, which did not interfere with fluorescence of the images. The ROIs used in this study is shown in Fig. 1. Due to the segmentation of certain tissues such as the episcleral tissue and ciliary body by the sectioning process, the different segments were analyzed individually and averaged.

### Statistical analysis

Unbalanced Analysis of Variance (ANOVA) and Levene’s test for homoscedasticity were performed for all ROIs, excluding the lens due to low sample size, in SAS (SAS Institute Inc., Cary, NC, USA) for the biodistribution experiment (α = 0.05). For lutein-loaded nanoparticle stability and release, statistical analysis was performed in Prism (GraphPad, San Diego, CA) using two-way ANOVA Tukey’s post hoc tests (α = 0.05) between different timepoints, formulations, and temperatures.

## Results

### Nanoparticle characterization

The average diameter, polydispersity index (PDI), and ζ-potential of Cy5 and lutein-loaded nanoparticles were measured by Dynamic Light Scattering (DLS) immediately after resuspension (Table 1). TEM imaging revealed spherical and well-dispersed lutein-loaded and Cy-5 labeled particles in the 200 nm size range in support of the DLS results (data not shown). Lutein entrapment efficiency was 87.6 ± 1.4%.

**Table 1:** Physical properties of lutein-loaded and Cy-5 labeled PLGA nanoparticles based on Dynamic Light Scattering.

Nanoparticle load	Average diameter (nm)	PDI	ζ-potential (mV)
*Lutein*	210.6 ± 3.3	0.119 ± 0.007	− 6.7 ± 0.3
*Cy-5*	241.7 ± 0.6	0.219 ± 0.015	− 14.1 ± 1.5

**Figure 2:**
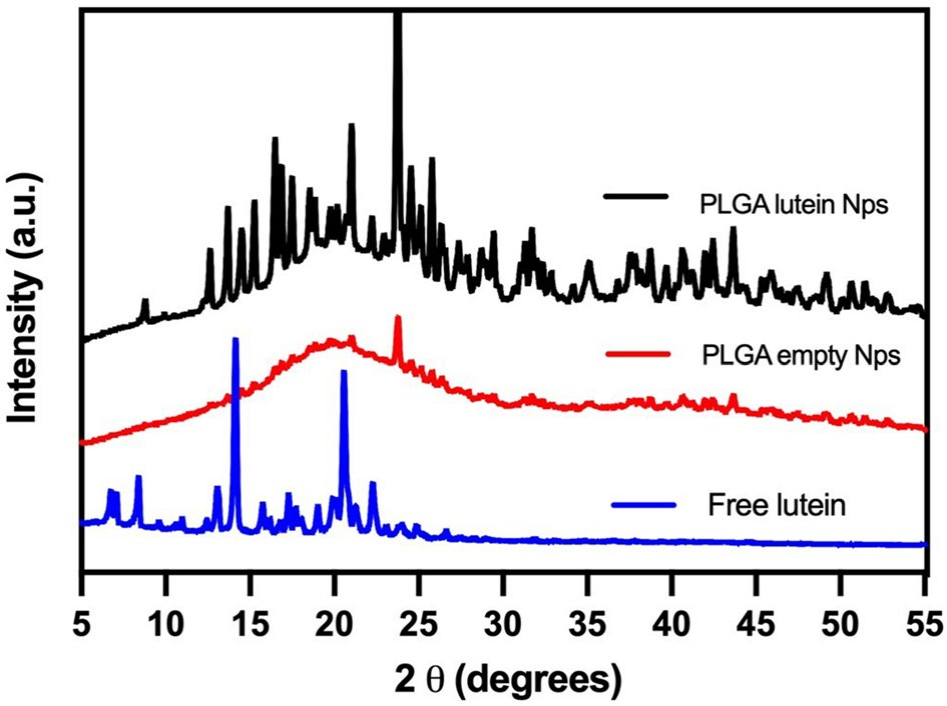
XRD spectrums of free lutein, empty PLGA NPs, and lutein-loaded PLGA NPs.

The XRD spectrums for lutein, empty PLGA NPs, and lutein-loaded PLGA NPs are shown in Fig. 2. The data for free lutein suggested a crystalline phase with diffraction angles at 6.76°, 7.08°, 8.39°, 14.09°, 20.57°. This pattern was observed for the lutein-loaded PLGA NPs suggesting that lutein dispersed in the polymeric matrix conserve its crystallinity.

### Physical stability of nanoparticles

**Figure 3:**
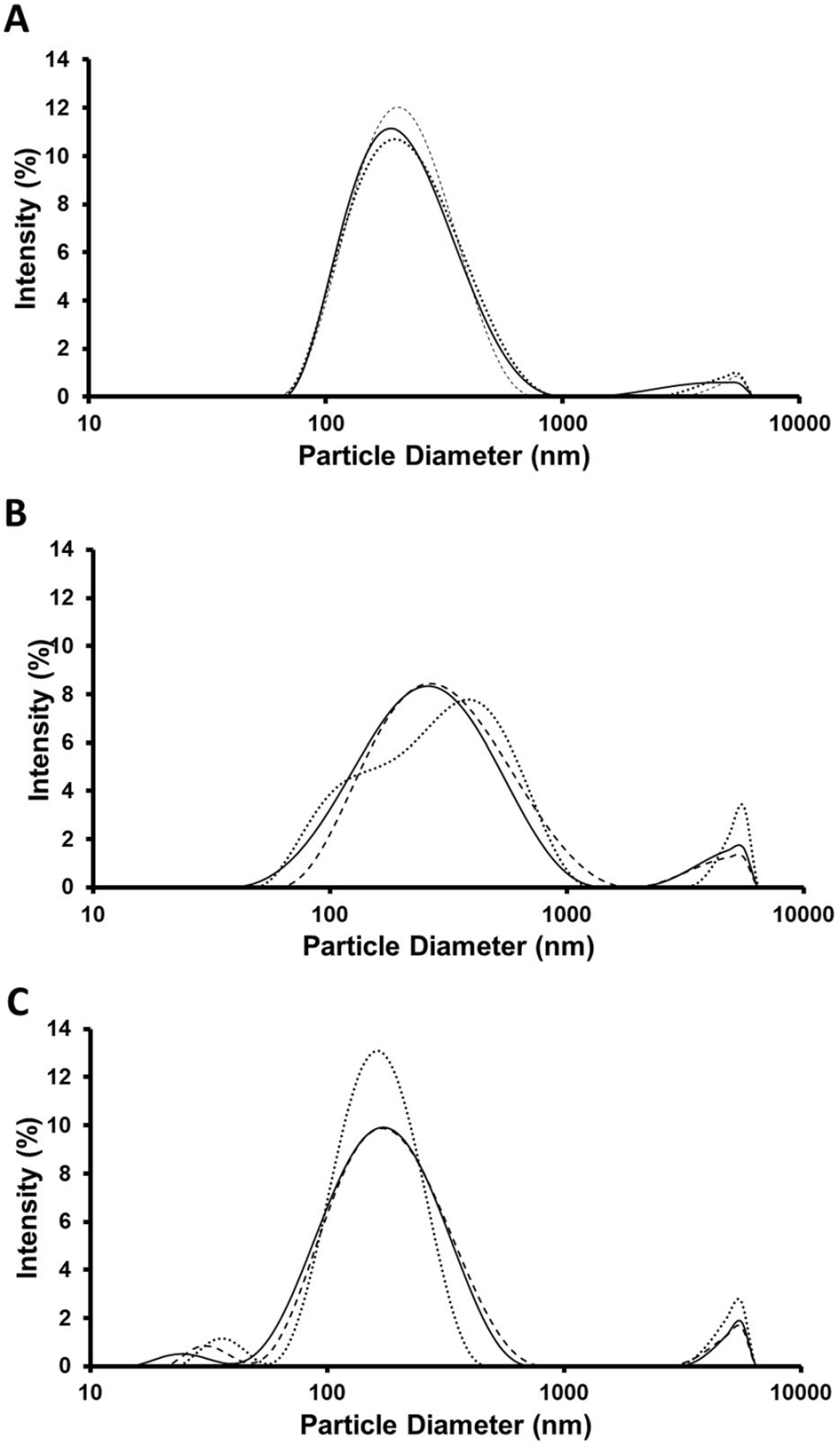
Size distribution plots for nanoparticles stored in suspension at 4 °C (**A**), 235 °C (**B**), and 37 °C (**C**) for 5 weeks.

Lutein-loaded nanoparticles in suspension stored at 4 °C, 25 °C, and 37 °C were analyzed via DLS after 5 weeks in suspension. According to the intensity curves, more nanoparticle aggregation occurred at 25 °C and 37 °C than at 4 °C based on the intensity of the secondary peaks in the higher size range (Fig. 3). Nanoparticles stored at 37 °C had an additional secondary peak in lower dimensions which likely represents micelles formed by dissociated surfactants (Fig. 3C).

**Figure 4:**
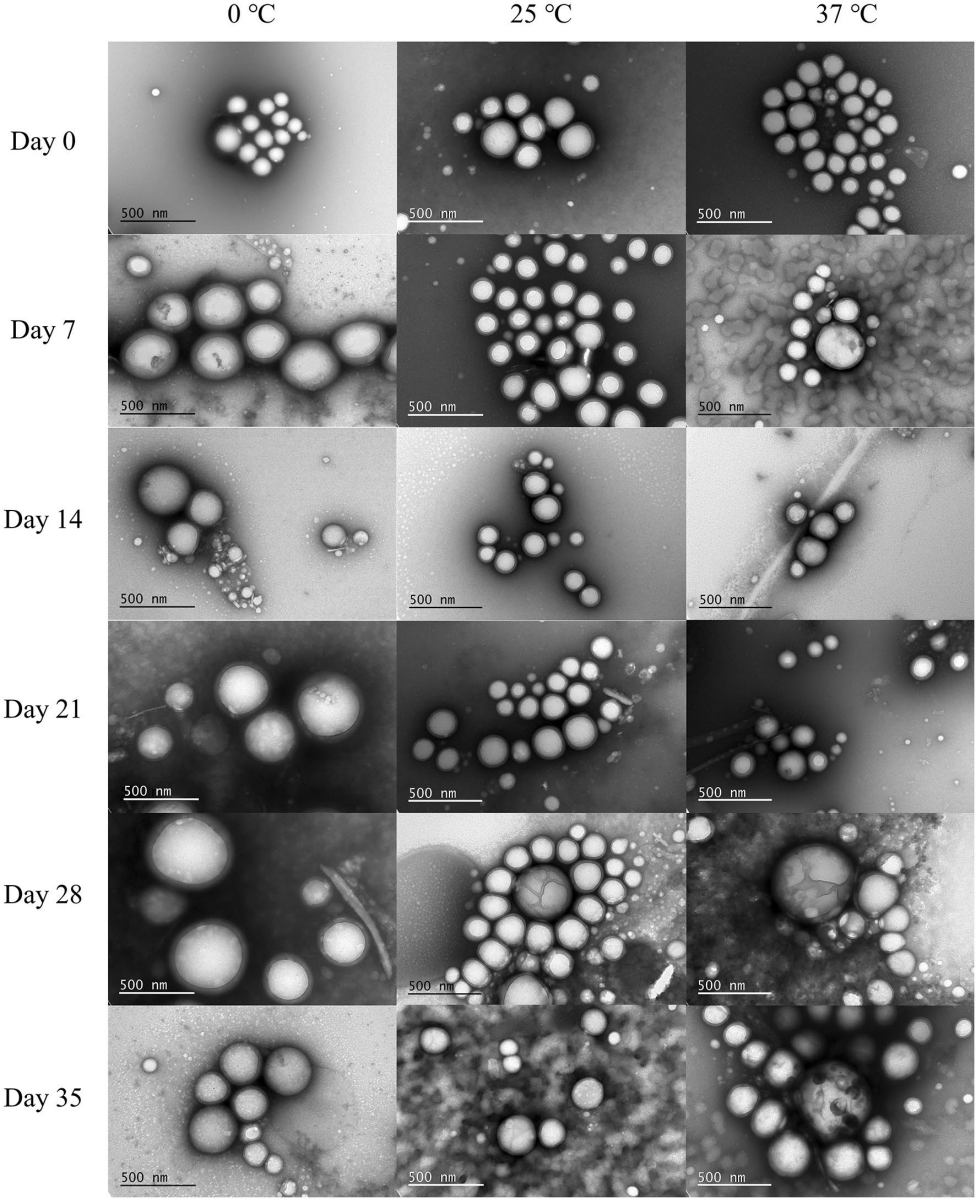
TEM micrographs (M = 100,000x) of lutein-loaded nanoparticles suspended for 5 weeks at different temperatures.

TEM did not show any apparent changes in the morphology of nanoparticles stored in suspension at 4 °C during the 5-week period; however, larger nanoparticles started to show signs of degradation by day 28 for nanoparticles stored at 25 °C and by day 7 for nanoparticles stored at 37 °C (Fig. 4). Slightly smaller and less defined structures were occasionally visible in samples stored at all temperatures, but were most apparent in the micrographs for 25 °C at day 35 and 37 °C as early as day 7 (Fig. 4). This comes in support of the DLS data, indicating that micelles were formed by dissociated surfactants from the nanoparticle surface under these conditions.

### Lutein release from nanoparticles

**Figure 5:**
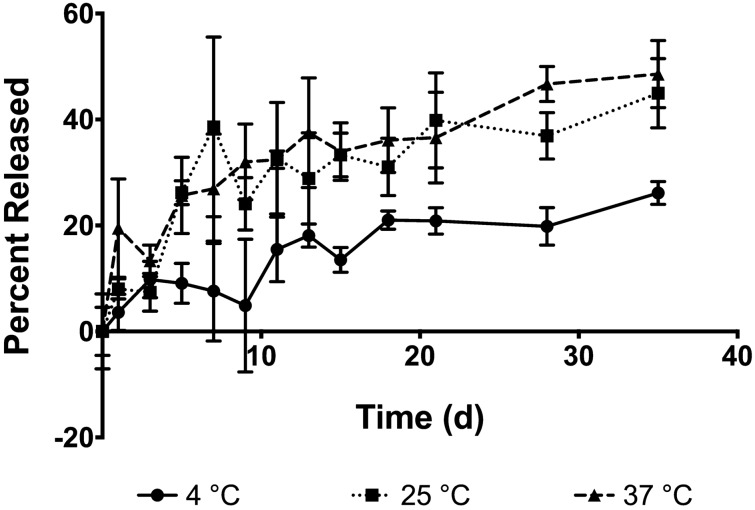
Lutein release from nanoparticles at 4 °C, 25 °C, 37 °C for 5 weeks. Release at 37 °C was significantly higher than release at 4 °C on day 5 and all following timepoints. Release at 25 °C was also significantly higher (p ≤ 0.05) than release at 4 °C on day 5 and all following timepoints except day 13 and 18. No significant differences exist between 25 °C and 37 °C.

Lutein release was measured over the course of 5 weeks at 4 °C, 25 °C, and 37 °C (Fig. 5). The release kinetics best fits a 0^th^ order trendline at all temperatures with the following k-values for 4 °C, 25 °C, and 37 °C respectively: 0.675, 1.0424, and 1.1038. By day 5, lutein release was significantly higher at 25 °C and 37 °C relative to 4 °C (Fig. 5). After 5 weeks of release, approximately 48% lutein had released at 37 °C, 44% at 25 °C, and only 26% at 4 °C.

#### Lutein thermal stability

**Figure 6:**
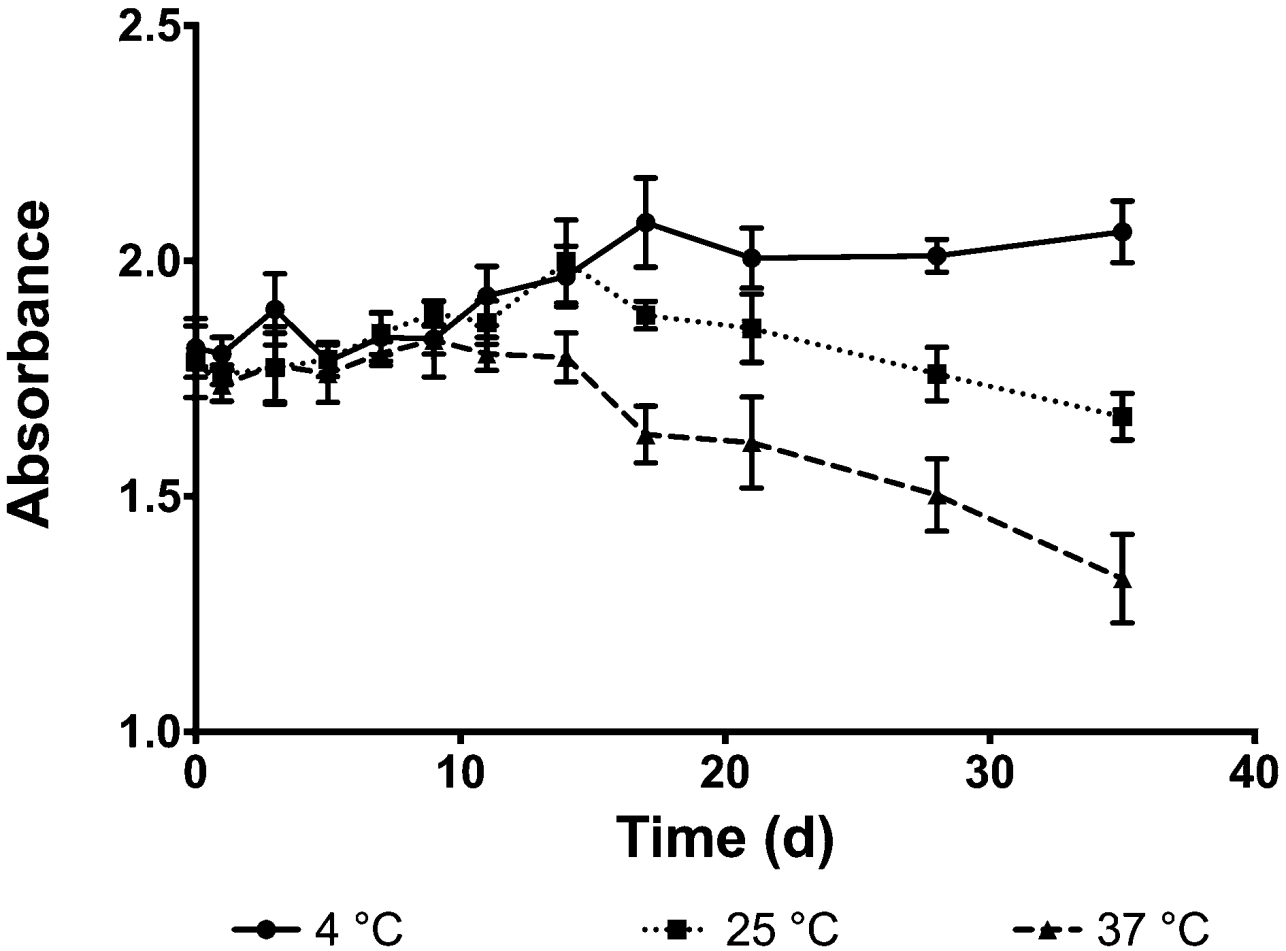
Degradation of lutein within nanoparticles at 4 °C, 25 °C, 37 °C. Absorbance is significantly lower, indicating lutein degradation, at 37 °C compared to other temperatures by day 14 and at 25 °C compared to 4 °C by day 17.

Lutein was protected from thermal degradation at 4 °C relative to the other temperatures (Fig. 6). Significantly more lutein degraded after 5 weeks at 37 °C than at the lower temperatures tested, and significantly more lutein degraded after 5 weeks at 25 °C than at 4 °C. Significant differences between groups were achieved at different times, between 37 °C and the other temperatures by day 14, and between 25 °C and 4 °C by day 17.

### Lutein photostability

**Figure 7:**
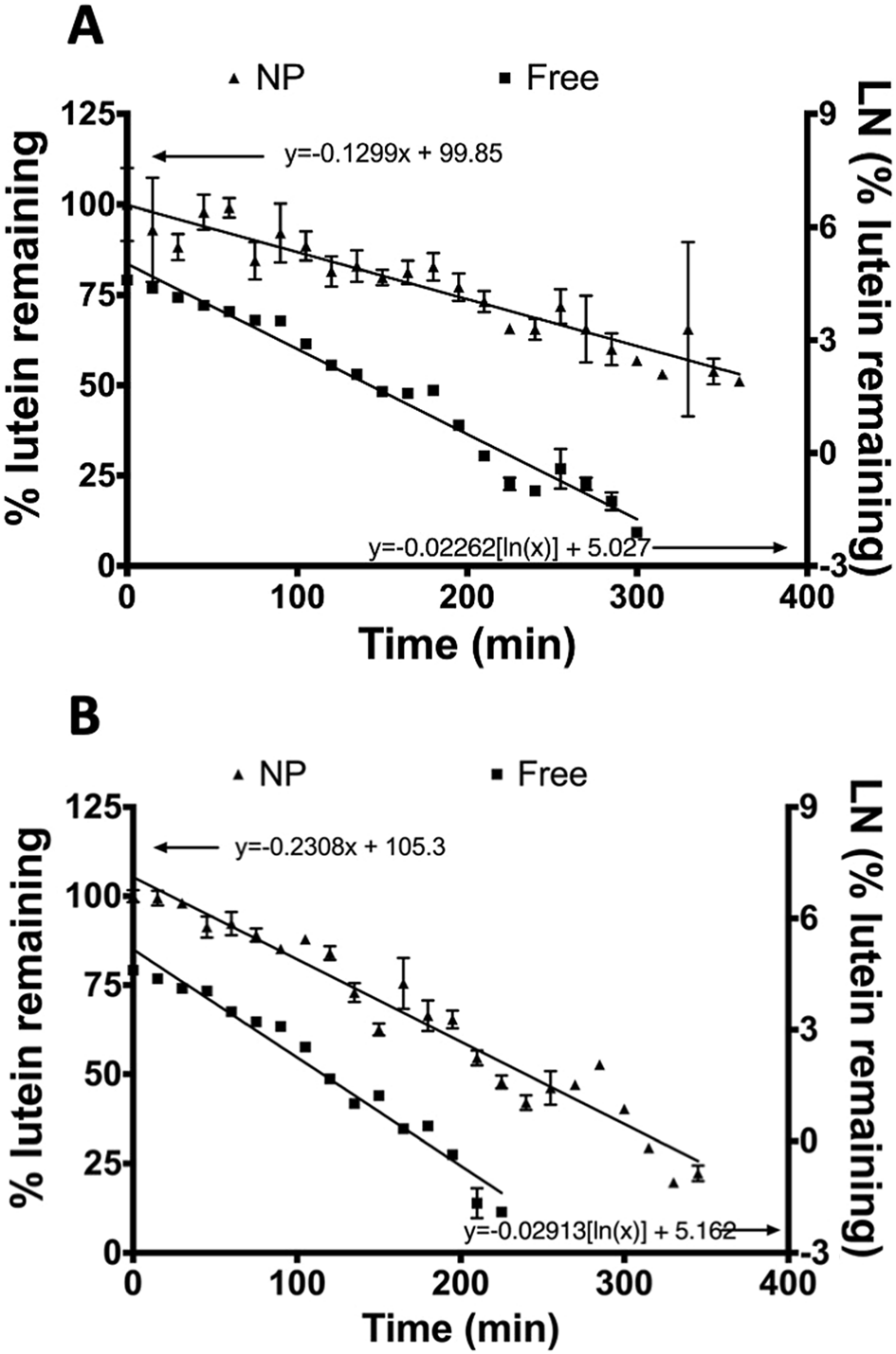
Lutein degradation under UV light exposure at (A) 25 °C (B) 37 °C. The percentage of Nano-entrapped lutein remaining, indicated by triangular data markers, correspond to the left axis, while the natural log of the percentage of free lutein remaining, indicated by square markers, corresponds to the right axis.

Under UV exposure, free lutein dissolved in DMSO degraded approximately fivefold faster than lutein loaded in nanoparticles at the same concentration. 50% lutein degraded within 1 h of exposure at 25 °C and 37 °C in free form, while it took 5 h for it to degrade at 37 °C and over 6 h at 25 °C when entrapped in nanoparticles. Free lutein degradation kinetics followed a 1^st^ order decrease with k-values 0.0226 ln[% lutein]/min at 25 °C and 0.02913 ln[% lutein]/min at 37 °C (Fig. 7). However, incorporation of lutein in nanoparticles shifted the degradation kinetics from 1^st^ order to 0^th^ order, with k-values 0.1299% lutein/min at 25 °C and 0.2308% lutein/min at 37 °C (Fig. 7).

### In vivo biodistribution

**Figure 8:**
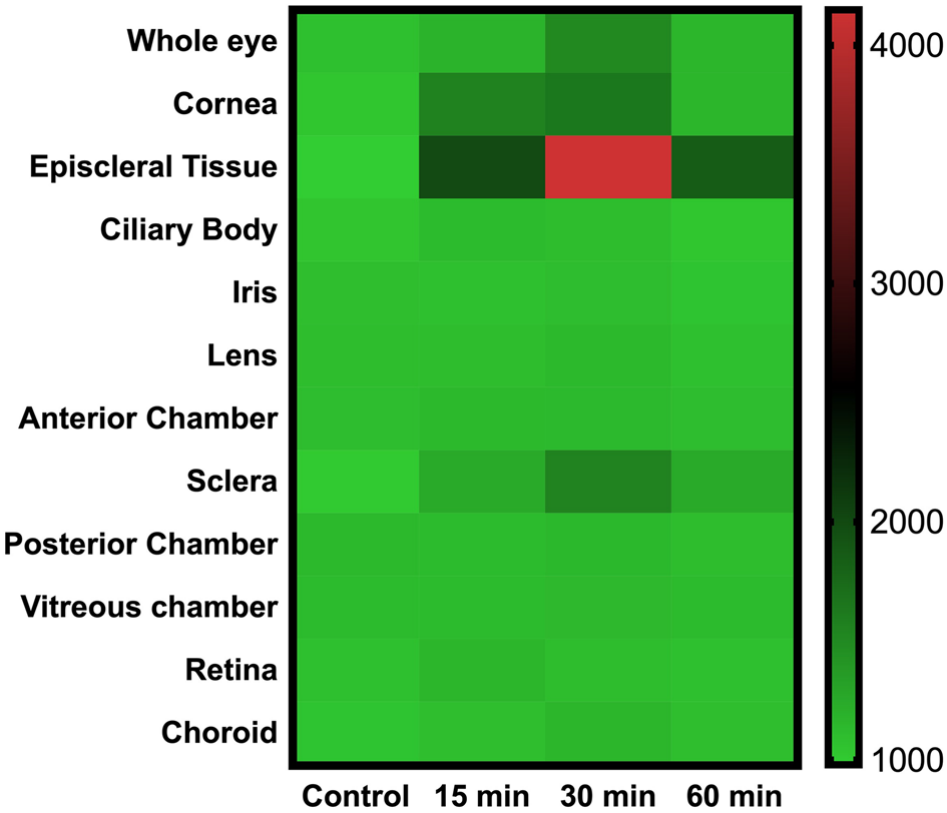
Heat map depicting average fluorescence intensity of each ROI. The average intensity of the whole eye in the 30 min treatment group is significantly higher (p ≤ 0.05) than all other timepoints and the control. The 30 min treatment group also had significantly higher average fluorescence than the control in the cornea, episcleral tissue, sclera, and choroid, and significantly higher average fluorescence than the 60 min treatment group in the choroid and episcleral tissue.

Results from the biodistribution pilot study conducted in mice are presented in supplementary material (Figs. S2–S6). Due to the high green autofluorescence of the eye tissues examined in the pilot study, the nanoparticles used in the full study were labeled with Cy-5, a far red fluorophore, instead of coumarin-6, a green fluorophore. In this full study, the average fluorescence intensity of the whole eye and ROIs in treated animals was compared against the control at different time points, 15, 30, and 60 min after treatment (Fig. 8). A significant increase in average fluorescence for the whole eye was observed at 30 min in the nanoparticle treated group compared to all groups. At the same time point, cornea and sclera had a significantly higher average fluorescence than the control. In the episcleral tissue and choroid, the 30 min group was significantly higher than the control and the 60 min group, but not the 15 min group. The Levene’s test (α = 0.05) p-values for episcleral tissue and sclera were significant (0.006 and 0.0173, respectively) indicating that at least one group had a significantly different variance than the others, while all other tissues had non-significant p-values indicating homoscedasticity, which is important for ANOVA accuracy.

A distinct but non-significant increase in average fluorescence intensity was observed when comparing the 15 min treatment group to the control in select tissues (cornea, episcleral tissue, sclera, and the whole eye section), followed by another increase at 30 min (significant in the whole eye section compared to 15 min), and a decrease at 1 h (significant in the whole eye, episcleral tissue, and choroid compared to 30 min) (Fig. 8).

It is important to note that not all tissues were equally represented in evaluated cryosections. The lens, in particular, was absent or fractured in most of the slides recovered and was therefore excluded from statistical analysis due to a low sample size. Artificial retinal detachment from sectioning was also common in the cryosections, though quantification was still performed by excluding the area of detachment from the ROI. The following sample sizes were used in each tissue for the control, 15 min, 30 min, and 60 min groups, respectively: whole eye (n = 8, 6, 10, 7), cornea (n = 8, 6, 10, 6), episcleral tissue (n = 8, 2, 10, 6), ciliary body (n = 8, 5, 9, 5), iris (n = 2, 4, 7, 4), lens (n = 2, 4, 2, 3), anterior chamber (n = 8, 6, 10, 4), sclera (n = 8, 6, 10, 7), posterior chamber (n = 7, 4, 5, 3) vitreous chamber (n = 8, 6, 10, 7), retina (n = 8, 6, 9, 6), and choroid (n = 7, 6, 9, 6).

## Discussion

The presence of a small secondary peak in the DLS size distribution plot for 37 °C in the 20–50 nm size range (Fig. 3) and the presence of small fluid-like particles observed in TEM images on day 7 at 37 °C and day 35 at 25 °C (Fig. 4) seem to indicate that a surfactant, most likely PVA, is dissociating from the nanoparticles when suspended at higher temperatures and forming micelles. The PVA micelles formed over time from dissociated surfactant may have impacted lutein release as free micelles in the suspension could facilitate the diffusion of lutein out of the nanoparticles. Since PLGA nanoparticles are bulk eroding^19^, degradation-related morphological changes were not apparent in the TEM images except in larger particles at higher temperatures (Fig. 4). Nanoparticles stored at 25 °C and 37 °C displayed a faster release profile than nanoparticles stored at 4 °C (Fig. 5).

When testing the thermal stability of lutein in nanoparticles, it was apparent that nano-entrapped lutein was relatively unstable at room temperature and physiological temperature compared to 4 °C, where no significant degradation occurred (Fig. 6). In addition to protecting lutein from thermal degradation, nanoparticles stored at 4 °C only released 26% lutein after five weeks while nearly half of it was released at 25 °C and 37 °C (Fig. 5). Nanoparticles significantly enhanced the photo-stability of lutein when exposed to UV light, slowing degradation compared to free lutein (Fig. 7). This indicates a photo-protective effect of nanoparticles on the entrapped lutein which may help extend the therapeutic potential of an ophthalmic lutein suspension after storage.

While the biodistribution pilot study had several limitations including low sample size and high autofluorescence of the tissue in the selected fluorescence marker’s emission range, there were nevertheless two important findings: a high degree of clearance occurred between the two measured timepoints, 30 min and 60 min, and incorporation of nanoparticles into a thermosensitive hydrogel did not appear to increase their uptake in the eyes of mice, possibly due to the mouse’s eyelids physically limiting contact of the hydrogel droplet with the eyes (Figs. S2–S6). We hypothesized that this would not be an issue in larger animal models. To address the limitations of the pilot study, a larger biodistribution study was performed using rats, an additional timepoint, a fluorescence marker that did not overlap with the tissues’ natural autofluorescence, and only nanoparticles suspended in saline due to their better performance in the pilot study. Additionally, the average fluorescence of each tissue was compared instead of the total fluorescence due to the varying sizes of tissues in the eye sections.

As expected for biodistribution associated with topical application, nanoparticle presence was most distinctive in the exterior eye tissues: cornea, episcleral tissue, and sclera (Fig. 8). The choroid was the only inner eye tissue with a treatment group significantly different than the control, and the difference was much smaller than in the exterior eye tissues, indicating limited penetration into the inner eye. The majority of topically applied substances in solution are subject to clearance by lacrimal fluids on the eye, thereby limiting bioavailability in the inner eye^20^ and may benefit from application of the nanoformulation in a thermosensitive gel^3,4,21^. Paracellular permeation of nanoparticles through the corneal epithelium is extremely challenging due to tight junctions between cells, forcing most nanoparticles to permeate via the transcellular route into the eye^22^. Thus, the corneal epithelium forms a strong barrier to hydrophilic particles. However, some nanoparticle formulation have been able to overcome the corneal barrier, such as super-cationic quantum dots which were induced the opening of epithelial tight junctions in the cornea and were effective in treating bacterial dermatitis in rabbits^23^.

The nanoparticles tested here likely reached the choroid via the periocular route, either directly through the sclera or first through the conjunctiva, a component of the episcleral tissue, and then through the sclera^24^. The latter pathway is supported as the likely route in this study due to the high uptake of nanoparticles by the episcleral tissue, providing them a means to avoid lacrimal elimination and penetrate the sclera. To reach the inner retina, nanoparticles in the choroid have to penetrate the outer BRB, formed by tight junctions between RPE cells^24^. As shown in Fig. 8, we did not observe an increase in the fluorescence in the retina possibly due to the inability of nanoparticles to penetrate the BRB or the low penetration into the inner eye following topical application. To enhance corneal penetration and bioavailability inside the eye, nanoparticle-based delivery systems should focus on extending the residence time of nanoparticles on the eye surface while simultaneously increasing transcellular permeation.

A clear trend was observed in terms of timing, where topically applied nanoparticles required more than 15 min after application to reach the peak concentration in these tissues but were mostly cleared within an hour (Fig. 8). It was not surprising that the most significant decreases from 30 min to 1 h occurred in the episcleral tissue and choroid, as both of these tissues contain dense vasculature and are responsible for rapid elimination of drugs in the eye^25,26^. Only about 20% lutein was released after 1 day at 37 °C, which appears to create an issue for the nanodelivery system’s ability to delivery lutein within the effective time frame before clearance. However the dialysis method used to assess release may not accurately represent in the in vivo release profile. It has been shown that diffusion of released compounds through the dialysis membrane may be the rate limiting step at higher temperatures where diffusion out of the nanoparticle is faster than that through the membrane, causing the measured release rate to appear slower than it actually is (Zambito et al. 2012). While certain modifications to the nanoparticle, such as increasing the glycolic acid ratio and decreasing the PLGA’s molecular weight, can increase the rate of release^19^, the greater focus should be on using strategies to attenuate the rate of clearance of nanoparticles from the eye. Such methods have been developed for other administration methods including sub-conjunctival injection by suspending nanoparticles in a thermosensitive hydrogel made of PLGA-PEG-PLGA copolymer and suprachoroidal injection by suspending nanoparticles in a non-Newtonian polymer matrix of carboxylmethylcellulose and methylcellulose for delivery to the ciliary body or hyaluronic acid for delivery to the choroid^25,26^. Additionally, the use of mucoadhesive coatings such as chitosan, gelatin, or PF68 may improve ocular penetration and reduce the rate of clearance by increasing corneal residence time, as it was found that core–shell nanoparticles with various mucoadhesive coatings persisted in the eye up to 4 h after topical application^27^.

For polymeric nanoparticles to be considered viable ocular drug delivery systems, further research is needed to assess their distribution profiles in the following various methods of administration for different nanoparticle compositions to determine what strategies are most effective for addressing specific ocular diseases.

## Conclusion

For an ophthalmic nanodelivery system to be used effectively in a home environment, its stability in an aqueous suspension must be known. Our work showed that storage of an aqueous, lutein-loaded nanoparticle suspension at 4 °C increases its shelf-life by delaying the release to only 26% in 5 weeks and enhancing the thermal stability of lutein compared to aqueous suspensions stored at 25 °C and 37°. Less than 30% lutein was released, no significant thermal degradation occurred, and little change was observed in the size, PDI, and morphology of nanoparticles after 5 weeks in the suspension kept at 4 °C. The most notable change was the decrease in ζ-potential between 0 and 4 days. Nanoparticles greatly enhanced the photo stability of lutein when exposed to UV light compared to free lutein solution.

### Animal use

Animal use and procedures in this study were approved by the Louisiana State University and A & M College (LSU A&M) Institutional Animal Care and Use Committee. The LSU A&M endorses and complies with the American Veterinary Medical Association (AVMA) position statement regarding animal welfare, and complies with the guidelines as stated in the National Institutes of Health’s (NIH) *Guide for the Care and Use of Laboratory Animals*, 8^th^ edition. This institution also endorses the position of the Association of American Veterinary Medical Colleges (AAVMC) regarding animal welfare, and complies with the provisions of the School of Veterinary Medicine, Division of Laboratory Animal Medicine (DLAM) Policy and Procedures Manual. In addition, this institution is fully accredited by AAALAC International, indicating verified compliance with the requirements for the proper care and treatment of all vertebrate laboratory animals, irrespective of species, location, investigator, use, or funding source. The University has on file with the Office for Protection from Research Risks (OPRR), an approved Assurance Statement (#A3612-01). The experimental protocols for this chapter were approved by the Louisiana State University Institution Animal Care and Use Committee (IACUC).

### Software used

- Prism 7.0 (GraphPad)
- SAS 9.4 (SAS Institute)
- Excel 2016 (Microsoft)
- Word 2016 (Microsoft)
- Powerpoint 2016 (Microsoft)
- Gen5 (Bioteck, Agilent).

## Supplementary Information

Supplementary Information.

## Data Availability

The datasets used and/or analyzed during the current study are available from the corresponding author on reasonable request.

## Abbreviations

ANOVA: Analysis of variance
AVMA: American Veterinary Medical Association
DLS: Dynamic light scattering
IACUC: Institutional Animal Care and Use Committee
LSU A&M: Louisiana State University and A&M
NP: Nanoparticles
PLGA: Poly(lactic-co-glycolic acid)
PVA: Polyvinyl alcohol
ROI: Region of interest
TEM: Transmission electron microscopy
