# Supplementary material for: Stability and ocular biodistribution of topically administered PLGA nanoparticles
Source: Sci Rep. 2021 Jun 10;11:12270. doi: 10.1038/s41598-021-90792-5 (PMC8192547; doi:10.1038/s41598-021-90792-5)
Supplement: Supplementary file 1 — Supplementary Information. [file 41598_2021_90792_MOESM1_ESM.pdf]

Stability and Ocular Biodistribution of Topically Administered PLGA Nanoparticles: Supplement

Sean Swetledge[1], Renee Carter[2], Rhett Stout[3], Carlos E. Astete[1], Jangwook P. Jung[1], Cristina M. Sabliov[4*]

[1]Department of Biological and Agricultural Engineering, Louisiana State University, Baton Rouge, Louisiana 70803, United States.

[2]Veterinary Clinical Sciences, Louisiana State University and LSU Veterinary Medicine, Skip Bertman Drive, Baton Rouge, Louisiana 70803, United States.

[3]Pathobiological Sciences, Louisiana State University and LSU Veterinary Medicine, Skip Bertman Drive, Baton Rouge, Louisiana 70803, United States.

[4]Department of Biological and Agricultural Engineering, Louisiana State University and LSU Agricultural Center, Baton Rouge, Louisiana 70803, United States. Electronic address: csabliov@agcenter.lsu.edu.

*Corresponding author

Biodistribution Pilot Study Methods

PLGA-PEG Copolymer Synthesis

PLGA-PEG copolymer was synthesized via N-hydroxysuccinimide (NHS) mediated amide coupling. The carboxyl terminal end of the PLGA was activated with NHS with using dicyclohexylcarbodiimide (DCC). PLGA (1,500 mg), DCC (10.4 mg), and NHS (5.8 mg) were added to a nitrogen flushed round bottom flask and dissolved in 25 ml anhydrous dichloromethane (DCM). The reaction solution was allowed to stir overnight at room temperature. Insoluble dicyclohexyl urea was filtered out with filter paper. The solution was concentrated in with a rotovapor until the polymer just began precipitating and forming a slurry. The PLGA-NHS was precipitated by adding dropwise to 200-300 mL cold anhydrous diethyl ether. After 15 min, the supernatant was removed and the polymer was allowed to dry under hood for 10 min. The polymer was then re-dissolved in 20 mL DCM, and the concentration and precipitation steps were repeated

for extra purity. The polymer was then dried via rotovapor, placed in a vacuum oven for 24 h, and stored at -20 °C.

NHS ester of the PLGA was conjugated with PEG (3 kDa) via amide linkage. PLGA-NHS (1 g) and 120 mg PEG were dissolved in extra dry DCM (15 mL) in a rubber sealed round bottom flask. The coupling reaction was performed over night at room temperature. The PLGA-PEG diblock copolymer was precipitated with cold methanol and purified with ethanol to remove excess PEG. The PLGA-PEG copolymer was then dried via rotovapor, placed in a vacuum oven for 24 h, and stored at -20°C until used in nanoparticle synthesis.

Nanoparticle Synthesis

Fluorescent nanoparticles were synthesized using the emulsion evaporation technique. A 2% w/v solution of PVA (31-50 kDa) was prepared in water by dissolving PVA at 60 °C. The organic phase was prepared by dissolving 400 mg PLGA (24-38 kDa) and 15 mg coumarin-6 in 8 ml ethyl acetate. The aqueous phase was a 100 ml solution of tween 80 (4.5 mg/ml) and was saturated with 10 ml ethyl acetate. The organic phase was added dropwise to the aqueous phase and allowed to stir for 10 min. The emulsion was passed through a microfluidizer 4 times at 30 kpsi. Ethyl acetate was evaporated out using a rotovapor for 90 min. Trehalose monohydrate was added (1100 mg) as a cryoprotectant, followed by 10 ml of the PVA solution. The nanoparticle suspension was frozen at -80 °C, freeze-dried for 48 h, and stored at -20 °C until use.

Nanoparticle Characterization

Freeze-dried nanoparticles were resuspended in water and analyzed by transmission electron microscopy (TEM) (Jeol 4000, Tokyo, Japan) and dynamic light scattering (DLS) using a Malvern Zetasizer (Malvern, United Kingdom) for size, polydispersity, and morphology.

Treatment Preparation

A thermosensitive hydrogel was prepared by dissolving 550 mg Poloxamer 407 and 40 mg Poly(ethylene glycol) (PEO) 1105 in water. The suspension was stirred for 24 h at low temperature for complete dissolution of the polymers. Free-dried nanoparticles were resuspended in either the thermosensitive hydrogel or phosphate buffered saline (PBS) (pH 7.4) at 20 mg/ml. Control treatments consisted of just the hydrogel or PBS.

Animals

Mice were divided into four groups based on harvesting timepoints and the nanoparticle treatment: 30 min PBS (n=2), 60 min PBS (n=3), 30 min hydrogel (n=4), 60 min hydrogel (n=3). One drop of the fluorescent nanoparticle preparation, approximately 12 µl, was applied to the left eye while holding the eye open for 30 s, and the same procedure was repeated with the PBS/hydrogel controls for the right eye. Animals were kept separated during the incubation period to prevent cross contamination of treatments between animals. After either 30 min or 60 min, animals were euthanized in a CO_2 gas chamber, and the eyes were enucleated, immersed in OCT

freezing media, and snap frozen in liquid nitrogen. Eyes were cryosectioned (10 μm), fixed on the slide with 2% w/v paraformaldehyde for 10 min, followed by 2 washes with PBS for 2 min. The slides were stained and preserved with DAPI containing mounting media (Permount), coverslipped, and imaged using a fluorescence microscope (BioTek Cytation 3).

Image Analysis

Fiji (ImageJ) software was used to quantify green fluorescence in the eye and individual structures of the eye: cornea, retina, and sclera. A threshold was applied to images to reduce background noise and filter out intense artifacts. Obvious artifacts such as dust outside the eye were cropped out as well as the lens due to intense green autofluorescence. Regions within the threshold were then quantified for average fluorescence and number of pixels, which where multiplied together to yield total fluorescence.

Statistical Analysis

Statistical analysis was performed in Prism (GraphPad), using two-way Anova post-hoc Tukey tests between experimental groups (α=0.05).

Biodistribution Pilot Study Results

Nanoparticle Characterization

The average diameter of the nanoparticles was 317 nm with a relatively high polydispersity index (PDI) of 0.468. The nanoparticles were slightly negatively charged with an average ζ-potential of -25±7.0. TEM micrographs show particles with little to no aggregation, likely due the hydrophilic PEG layer (Figure S1.).

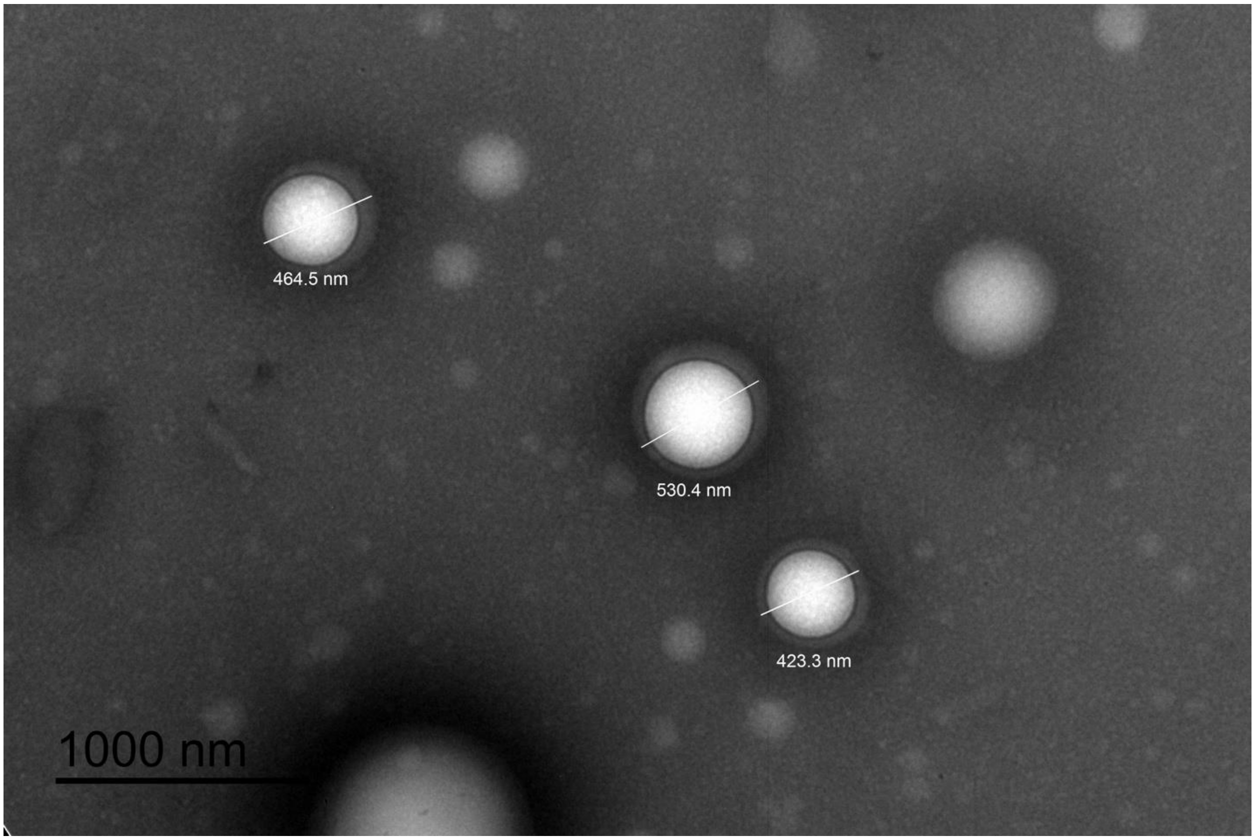

Figure S1. TEM micrograph (M=40,000x) of coumarin-6 loaded PLGA-PEG nanoparticles.

Image Analysis

Image analysis was done to remove as much of the background noise and autofluorescence as possible and quantify the amount of fluorescence in the eye. In general, fluorescence intensity was higher 30 min after treatment than after 60 min with animals treated with nanoparticles suspended in PBS showing higher intensity than animals treated with nanoparticles suspended in the hydrogel (Figure S2.). Fluorescence data specifically in the cornea showed relatively consistent results (Figure S3.), while the values measured in the retina and sclera were much more variable (Figure S4, S5.). In both the cornea and the entire eye, fluorescence was significantly higher in animals treated with nanoparticles suspended in saline after 30 min compared to the control ($p \leq 0.05$). No other statistically significant differences were measured.

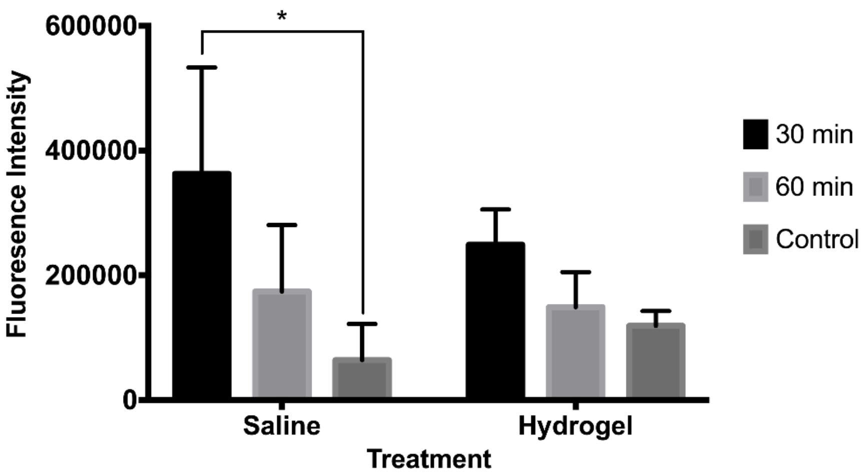

Figure S2. Fluorescence intensity in the entire eye region. 30 min saline (n=2), 60 min saline (n=3), saline control (n=3), 30 min hydrogel (n=4), 60 min hydrogel (n=3), hydrogel control (n=4). *p≤0.05.

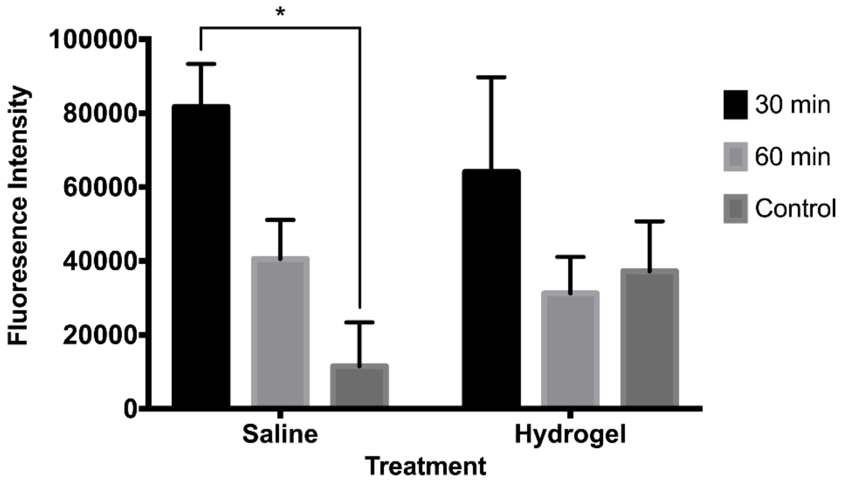

Figure S3. Fluorescence intensity in the cornea region. 30 min saline (n=2), 60 min saline (n=3), saline control (n=3), 30 min hydrogel (n=4), 60 min hydrogel (n=3), hydrogel control (n=4). *p≤0.05.

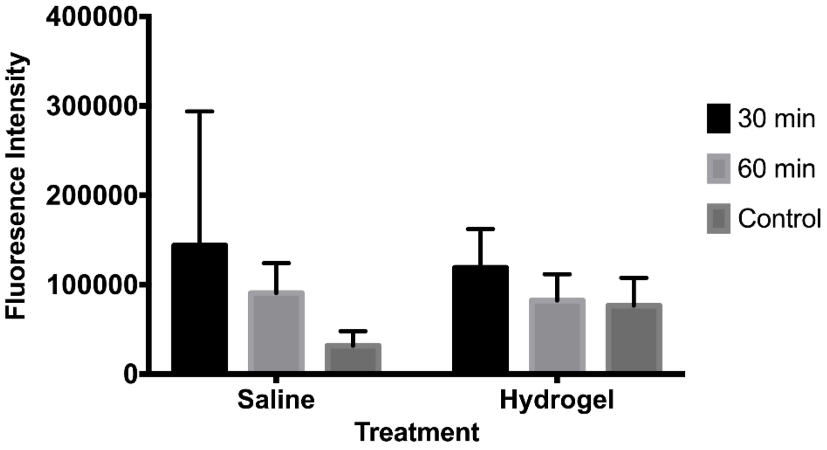

Figure S5. Fluorescence intensity in the sclera region. 30 min saline (n=2), 60 min saline (n=3), saline control (n=3), 30 min hydrogel (n=4), 60 min hydrogel (n=3), hydrogel control (n=4). *p≤0.05.

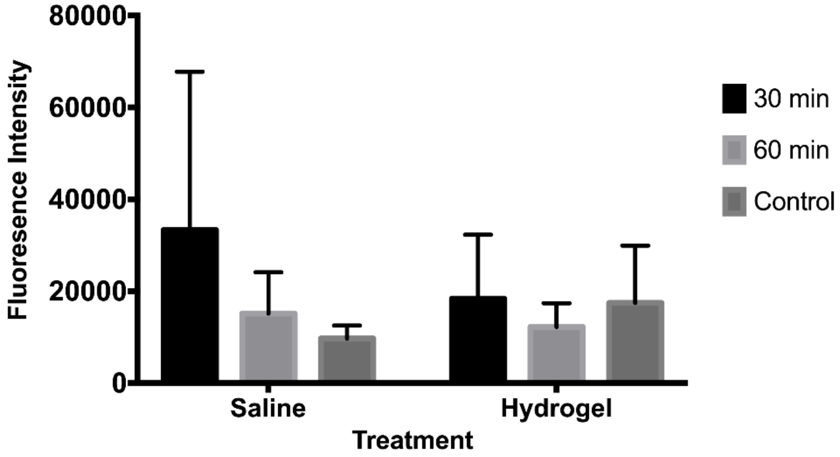

Figure S6. Fluorescence intensity in the retina region. 30 min saline (n=2), 60 min saline (n=3), saline control (n=3), 30 min hydrogel (n=4), 60 min hydrogel (n=3), hydrogel control (n=4). *p≤0.05.
